# Vitamin A decreases pre-receptor amplification of glucocorticoids in obesity: study on the effect of vitamin A on 11beta-hydroxysteroid dehydrogenase type 1 activity in liver and visceral fat of WNIN/Ob obese rats

**DOI:** 10.1186/1475-2891-10-70

**Published:** 2011-06-23

**Authors:** Vara Prasad SS Sakamuri, Prashanth Ananthathmakula, Giridharan Nappan Veettil, Vajreswari Ayyalasomayajula

**Affiliations:** 1 Department of Biochemistry, National Institute of Nutrition, Indian Council of Medical Research, Jamai Osmania PO, Hyderabad-500 604, Andhra Pradesh, India; 2 National Center for Laboratory Animal Sciences, Indian Council of Medical Research, Jamai Osmania PO, Hyderabad-500 604, Andhra Pradesh, India

## Abstract

**Background:**

11β-hydroxysteroid dehydrogenase type 1 (11β-HSD1) catalyzes the conversion of inactive glucocorticoids to active glucocorticoids and its inhibition ameliorates obesity and metabolic syndrome. So far, no studies have reported the effect of dietary vitamin A on 11β-HSD1 activity in visceral fat and liver under normal and obese conditions. Here, we studied the effect of chronic feeding of vitamin A-enriched diet (129 mg/kg diet) on 11β-HSD1 activity in liver and visceral fat of WNIN/Ob lean and obese rats.

**Methods:**

Male, 5-month-old, lean and obese rats of WNIN/Ob strain (n = 16 for each phenotype) were divided into two subgroups consisting of 8 rats of each phenotype. Control groups received stock diet containing 2.6 mg vitamin A/kg diet, where as experimental groups received diet containing 129 mg vitamin A/Kg diet for 20 weeks. Food and water were provided *ad libitum*. At the end of the experiment, tissues were collected and 11β-HSD1 activity was assayed in liver and visceral fat.

**Results:**

Vitamin A supplementation significantly decreased body weight, visceral fat mass and 11β-HSD1 activity in visceral fat of WNIN/Ob obese rats. Hepatic 11β-HSD1 activity and gene expression were significantly reduced by vitamin A supplementation in both the phenotypes. CCAAT/enhancer binding protein α (C/EBPα), the main transcription factor essential for the expression of 11β-HSD1, decreased in liver of vitamin A fed-obese rats, but not in lean rats. Liver × receptor α (LXRα), a nuclear transcription factor which is known to downregulate 11β-HSD1 gene expression was significantly increased by vitamin A supplementation in both the phenotypes.

**Conclusions:**

This study suggests that chronic consumption of vitamin A-enriched diet decreases 11β-HSD1 activity in liver and visceral fat of WNIN/Ob obese rats. Decreased 11β-HSD1 activity by vitamin A may result in decreased levels of active glucocorticoids in adipose tissue and possibly contribute to visceral fat loss in these obese rats. Studying the role of various nutrients on the regulation of 11β-HSD1 activity and expression will help in the evolving of dietary approaches to treat obesity and insulin resistance.

## Background

11β-hydroxysteroid dehydrogenase type 1 (11β-HSD1) modulates the local glucocorticoid metabolism by catalyzing the conversion of inactive glucocorticoids (cortisone in humans and 11-dehydrocorticosterone in rodents) to active glucocorticoids (cortisol in humans and corticosterone in rodents). Recent studies have shown that, 11β-HSD1 plays an important role in the development of obesity [1,2]. Transgenic mice overexpressing 11β-HSD1 in adipose tissue developed visceral obesity [3], where as 11β-HSD1 knock-out mice displayed resistance to diet-induced obesity [4]. Administration of carbenoxolone, a non-selective 11β-HSD1 inhibitor, has been shown to decrease obesity in animal models [5]. 11β-HSD1 gene expression is directly regulated by CCAAT/enhancer-binding protein α (C/EBPα) [6], where as its expression is down regulated by Liver × receptor α (LXRα) [7].

Studies on rodent models of obesity provided valuable information regarding the molecular mechanisms involved in the development of obesity and its associated complications. WNIN/Ob obese rat colony was established by selective breeding of spontaneously mutated obese rat, identified in 80 year-old inbred wistar rat colony at National Institute of Nutrition (NIN, Hyderabad, India) [8]. The rat colony shows three phenotypes (also genotypes), lean (+/+), carrier (+/-) and obese (-/-). WNIN/Ob lean and carrier rats are fertile, where as obese rats are sterile. The obese rat colony is maintained by crossing between carrier rats, which results in genotypic ratio (and also phenotypic ratio) of 1:2:1 (lean: carrier: obese) indicating the autosomal co-dominant nature of the mutation. WNIN/Ob obese rats develop severe obesity with onset around 35 days of age. These rats are hyperphagic, hyperinsulinemic, hyperleptinemic and dyslipidemic [8]. Preliminary studies on WNIN/Ob obese rats have revealed no molecular defects in leptin and leptin receptor. Recent studies have located the mutation on 5^th ^chromosome [unpublished data] and further investigations are underway to identify the molecular mutation leading to the development of obesity in these obese rats.

Diet plays an important role in the development, progression and amelioration of chronic diseases like obesity and diabetes. Studies on the effect of nutrients on 11β-HSD1 activity and expression will greatly help in understanding the mechanisms underlying the development of obesity and its associated metabolic disorders. Vitamin A, an important micronutrient has divergent biological functions in animal physiology. Vitamin A-enriched diet has been shown to decrease adiposity and alter adipose tissue gene expression in rats [9]. Our previous studies on supplementation of high but non-toxic dose of vitamin A to obese rats of WNIN/Ob strain have shown to decrease adiposity [10,11]. So far, no studies have addressed the role of vitamin A in the regulation of 11β-HSD1 activity in normal and obese conditions. As vitamin A is known to alter the expression of genes regulated by C/EBPα [12], here, we hypothesize that supra-physiological (high but non-toxic) dose of vitamin A modulates 11β-HSD1 activity in adipose tissue under normal and obese conditions. To test our hypothesis, we studied the activity of 11β-HSD1 in visceral fat and liver of WNIN/Ob lean and obese rats, after chronic challenging with vitamin A-enriched diet (129 mg/Kg diet) for 20 weeks.

## Methods

### Animal experiment

Male, 5-month-old WNIN/Ob lean and obese rats (n = 16 for each phenotype) were obtained from the National Centre for Laboratory animal sciences, and divided into two groups A and B based on phenotype. Each group was further divided into two subgroups (AI, AII & BI, BII) consisting of 8 rats from each phenotype. The animals were housed individually in a temperature (22 ± 2°C) and light controlled (12 h cycle) animal facility. Animals of AI and BI subgroups were fed on stock diet containing 2.6 mg of vitamin A/Kg diet (Recommended daily allowance), while those in AII and BII subgroups were fed on experimental diet containing 129 mg of vitamin A/Kg diet (as retinyl palmitate- a generous gift from Nicholas Piramal India Limited). Animals were maintained on their respective diets for a period of 20 weeks. Food and water were provided ad libitum. Daily food intake and weekly body weights were recorded. The study was approved by Institutional Animal Ethical Committee (IAEC). At the end of the experiment, blood was collected after overnight fast, between 9.00 AM and 11.00 AM. Animals were sacrificed and tissues were collected and stored at -80°C for further analysis.

### Plasma and tissue parameters

Plasma corticosterone levels were measured by radioimmunoassay (RIA) kit method (Siemens, Los Angeles, USA). Plasma, liver and visceral fat retinol levels were measured by methods described previously [10].

### Measurement of 11β-HSD1 activity

11β-HSD1 functions as a reductase *in vivo*, reactivating corticosterone from inactive 11-dehydrocorticosterone. However, in tissue homogenates, dehydrogenase activity predominates, therefore, 11β-HSD1 activity was measured by the conversion of corticosterone to 11-dehydrocorticosterone. We studied 11β-HSD1 activity in visceral fat by taking omental adipose tissue, as it is known to exhibit higher enzyme activity. Post-nuclear fractions from liver and visceral fat were prepared by centrifuging tissue homogenate at 1000 g for 20 min. 11β-HSD1 activity was measured in post nuclear fractions of liver and visceral fat by incubating in duplicate at 37°C in Krebs-Ringer buffer containing glucose (0.2%), NADP (1 mM) and 1, 2, 6, 7-[^3^H_4_] corticosterone (50 nM). Conditions were optimized for both tissues to ensure first order kinetics, by adjusting protein concentrations for liver (40 μg/ml) and omental adipose tissue (1 mg/ml). After incubation (40 min for liver and 6 h for omental adipose tissue), steroids were extracted with ethyl acetate, the organic phase was evaporated under nitrogen, and extracts resuspended in mobile phase (50% water, 30% acetonitrile and 20% methanol). Steroids were separated by HPLC using reverse phase C18 column and fractions containing corticosterone and 11-dehydrocorticosterone were collected and radioactivity was counted in liquid scintillation counter (PerkinElmer, USA).

### Measurement of glycerol-3-phosphate dehydrogenase (GPDH) activity

50 mg of liver tissue was homogenized in 10 mM Tris-Hcl buffer (pH-7.4) containing 1 mM 2-mercaptoethanol and 5% protease inhibitor cocktail (Sigma, USA). Homogenate was centrifuged at 10,000 g for 20 minutes at 4°C and the supernatant was used for the estimation of cytosolic GPDH activity. GPDH activity was measured by the method of White and Kaplan and expressed as nanomoles of NADH utilized/min/mg of protein (13).

### Measurement of relative gene expression by semi-quantitative Reverse Transcription PCR

As lean rats have limited omental adipose tissue, which we utilized for the enzyme assay, we carried out gene expression work only in liver but not in adipose tissue. Total cellular RNA from liver was extracted using the method of Chomczynski and Sacchi [14]. The integrity of RNA was checked using 1% agarose gels stained with ethidium bromide. Total RNA was quantified by spectrophotometric absorption at 260 nm. 1.0 μg of RNA was used for synthesizing first strand cDNA. The reverse transcription (RT) reaction was carried out by incubating RNA with 0.5 μg oligo dT primer (Sigma, USA) and 100 units of Molony murine leukemia virus reverse transcriptase (Finnzymes, Espoo, Finland) at 37°C for 60 min. Total reaction volume used for RT was 20 μL. An aliquot of cDNA was amplified in a 20 μL reaction mixture. PCR conditions are given below: denaturation at 94°C for 1 minute, annealing at 60-64°C for 45 sec and polymerization for 70 °C for 1 min with DyNAzyme II DNA polymerase (Finnzymes, Espoo, Finland). A final extension was carried out at 70 °C for 7 min. The amount of RNA and the annealing temperature for different genes were standardized for linearity. Sequences of primers used for amplification are 11β-HSD1: forward primer-5'-GAGGAAGGGCTCCAG-3' and reverse primer-5'-GAGCAAACTTGCTTGCA-3'(NM_017080), LXRα: forward primer-5'-GCCCCATGGACACCTA-3' and reverse primer-5'-TGAGGGTCGGGTGCAA-3' (NM_031627), C/EBPα: forward primer-5'-GAGCCGAGATAAAGCCAA-3' and reverse primer 5'-CTTTCAGGCGACACCA-3' (NM_012524), β-actin: forward primer-5'- ACCAACTGGGACGACATGGA-3' and reverse primer-5'- TCTCAAACATGATCTGGGTCA-3' (NM_031144). β-actin mRNA was amplified as an internal control. Number of amplification cycles for each gene was standardized so that amplification will be in the logarithmic phase. After amplification, 8 μL of reaction mixture were electrophoresed on agarose gel (2%) in Tris-acetate EDTA buffer (pH 8.2). The ethidium bromide stained bands were visualized by UV-transilluminator and analyzed densitometrically using Quantity One software (Bio-Rad, version 4.4.0).

### Immunoblotting

Nuclear protein was extracted from liver as described previously [15,16]. Protein content was estimated by Bradford method. Proteins were separated by 10% SDS-PAGE gel and probed by specific antibodies raised against C/EBPα and LXRα (Santa Cruz Biotechnology, Inc., and Santa Cruz, CA). Equal loading of protein and transfer were ensured by staining membranes with Ponseau S. Bands were scanned using GS 710 densitometer (Biorad, CA, USA) and band densities were analyzed by Quantity one software (Biorad, version 4.4.0).

### Statistics

Data were analyzed by one way ANOVA by using least significant difference (LSD) post hoc test. Data are presented as mean ± S.E. Statistical significance was taken at P < 0.05 level (two tailed).

## Results

### Effect of vitamin A supplementation on physical and biochemical parameters

Vitamin A supplementation significantly reduced body weight, body weight gain and visceral fat mass in obese rats compared to stock diet-fed obese rats (Table 1). Vitamin A supplementation did not alter the visceral fat mass or body weight in lean rats as compared with their stock-diet fed counterparts (Table 1). Food intake was not affected by vitamin A supplementation in both the phenotypes as compared with their respective control groups (Table 1). Consumption of vitamin A-enriched diet for 20 weeks significantly increased retinol levels in liver and visceral fat of lean and obese rats as compared with their respective stock-diet fed rats (Table 2). Plasma corticosterone levels were not altered by vitamin A supplementation in both the phenotypes (Table 2).

**Table 1:** Effect of vitamin A supplementation on food intake and physical parameters in WNIN/Ob lean and obese rats

	AI	AII	BI	BII
Pre-treatment body weight (g)	375 ± 16	362 ± 25	628 ± 38	590 ± 11

Post-treatment body weight (g)	406 ± 23	452 ± 15	916 ± 48	778 ± 19*

Body weight (g)	91 ± 20	89 ± 14	285 ± 19	188 ± 14#

Daily food intake (g)	18 ± 0.6	18 ± 0.8	26 ± 1.4	26 ± 0.9

Visceral fat (g/100 g body.wt)	2.3 ± 0.4	2.5 ± 0.4	10.5 ± 0.7	7.0 ± 0.5#

Values are mean ± S.E of 8 rats. Values with ^# ^and * mark are significant at P < 0.01 and < 0.05 level respectively (by one-way ANOVA). Comparisons were made between normal and high vitamin A-fed groups of each phenotype (AI-Lean: 2.6 mg vitamin A/Kg diet, AII-Lean: 129 mg vitamin A/Kg diet, BI-Obese: 2.6 mg vitamin A/kg diet and BII-Obese: 129 mg vitamin A/kg diet)

**Table 2:** Effect of vitamin A supplementation on biochemical parameters in WNIN/Ob lean and obese rats

	AI	AII	BI	BII
Plasma corticosterone (ng/mL)	227 ± 50	170 ± 15	256 ± 27	287 ± 34

Plasma retinol (μg/dL)	28 ± 0.5	28 ± 0.5	40 ± 0.6	43 ± 2.0

Liver total retinol (mg/g tissue)	0.9 ± 0.1	9.8 ± 0.4^##^	0.4 ± 0.02	8.1 ± 0.3^##^

Visceral fat retinol (μg/g tissue)	4.0 ± 1.2	41 ± 2.7^##^	2.0 ± 0.1	4.8 ± 2.0^##^

Values are means ± S.E of 8 rats. Mean values with^## ^mark are significant at P < 0.001 level (by one-way ANOVA). Comparisons were made between normal and high vitamin A-fed groups of each phenotype (AI-Lean: 2.6 mg vitamin A/kg diet, AII-Lean: 129 mg vitamin A/kg diet, BI-Obese: 2.6 mg vitamin A/kg diet and BII-Obese: 129 mg vitamin A/kg diet)

### Effect of vitamin A supplementation on 11β-HSD1 activity in visceral fat

**Figure 1:**
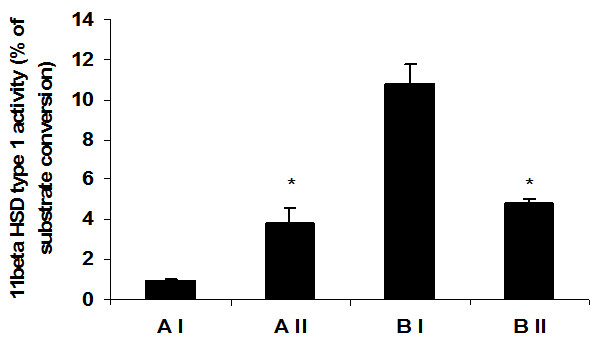
**Effect of vitamin A supplementation on adipose tissue 11β-HSD1 activity in WNIN/Ob lean and obese rats**. Values are means ± S.E for 8 rats. Mean values with * mark are significant at P ≤0.05 level (by one-way ANOVA). Comparisons were made between normal and high vitamin A-fed groups of each phenotype (AI-Lean: 2.6 mg vitamin A/kg diet, AII-Lean: 129 mg vitamin A/kg diet, BI-Obese: 2.6 mg vitamin A/kg diet and BII-Obese: 129 mg/kg diet).

Vitamin A supplementation significantly decreased 11β-HSD1 activity in visceral fat of obese rats as compared to stock diet-fed obese rats (Figure 1). In contrast to the observation in obese rats, vitamin A supplementation significantly increased 11β-HSD1 activity in visceral fat of lean rats as compared with their stock diet-fed lean counterparts (Figure 1).

### Effect of vitamin A supplementation on hepatic 11β-HSD1 activity and expression

**Figure 2:**
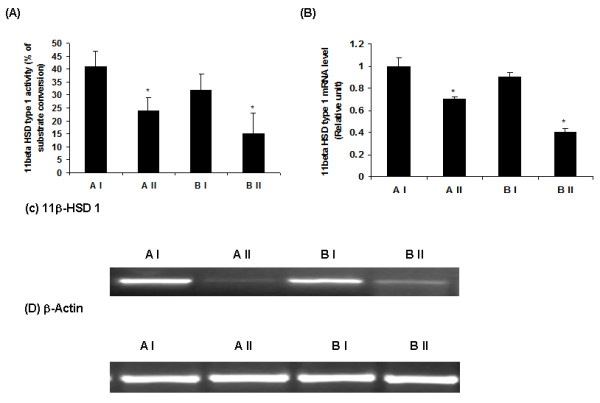
**(A) Effect of vitamin A supplementation on hepatic 11β-HSD1 activity in WNIN/Ob lean and obese rats**. Values are means ± S.E for 8 rats. Mean values with * mark are significant at P ≤0.05 level (by one-way ANOVA). (B) Effect of vitamin A supplementation on 11β-HSD1 gene expression in liver of WNIN/Ob lean and obese rats. Quantified densitometric values are expressed relative of 1 for lean control rats (AI). (C) Representative photo of 11β-HSD1 PCR product, stained by ethidium bromoide. (D) Representative photo of PCR β-Actin product (internal control), stained by ethidium bromide. Comparisons were made between normal and high vitamin A-fed groups of each phenotype (AI-Lean: 2.6 mg vitamin A/kg diet, AII-Lean: 129 mg vitamin A/kg diet, BI-Obese: 2.6 mg vitamin A/kg diet and BII-Obese: 129 mg/kg diet).

Vitamin A supplementation significantly decreased 11β-HSD1 activity in the liver of lean and obese rats as against their stock diet-fed lean and obese counterparts (Figure 2). In line with the decreased enzyme activity, 11β-HSD1 mRNA levels were significantly lower in high vitamin A-fed lean and obese rats as compared with their stock diet-fed lean and obese counterparts (Figure 2).

### Effect of vitamin A supplementation on C/EBPα, LXRα mRNA and protein levels in liver

**Figure 3:**
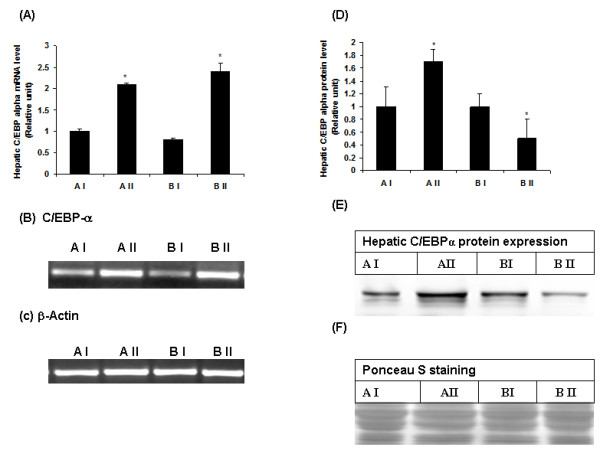
**(A) Effect of vitamin A supplementation on hepatic C/EBPα mRNA and protein levels in WNIN/Ob lean and obese rats**. Quantified densitometric values are expressed relative of 1 for lean control rats (AI). (B) Representative photo of C/EBPα PCR product, stained by ethidium bromoide. (C) Representative photo of β-Actin PCR product (internal control), stained by ethidium bromide. (D) Quantified densitometric values from the western blot are expressed relative of 1 for A-I (lean) as control. (E) Representative Western blot of hepatic C/EBPα protein. (F) Ponseau stained western blot. Values are means ± SE for 3-4 rats at P ≤0.05 level. Comparisons were made between normal and high vitamin A-fed groups of each phenotype (AI-Lean: 2.6 mg vitamin A/kg diet, AII-Lean: 129 mg vitamin A/kg diet, BI-Obese: 2.6 mg vitamin A/kg diet and BII-Obese: 129 mg/kg diet).

**Figure 4:**
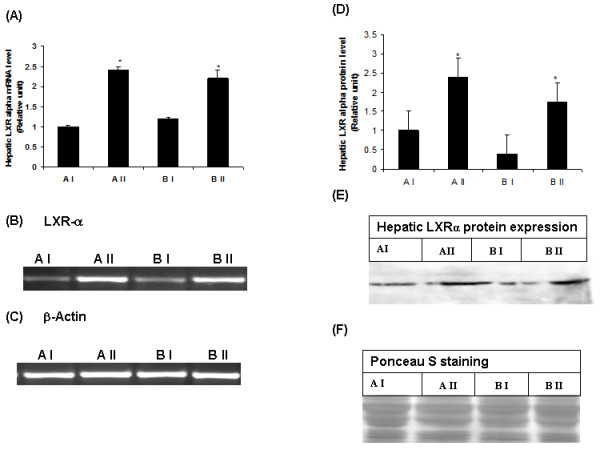
**(A) Effect of vitamin A supplementation on hepatic LXRα mRNA and protein levels in WNIN/Ob lean and obese rats**. Quantified densitometric values are expressed relative of 1 for AI (lean) as control. (B) Representative photo of LXRα PCR product, stained by ethidium bromoide. (C) Representative photo of β-Actin PCR product (internal control), stained by ethidium bromide. (D) Quantified densitometric values from the western blot are expressed relative of 1 for lean control rats (AI). (E) Representative Western blot of hepatic LXRα protein. (F) Ponseau stained western blot. Values are means ± SE for 3-4 rats at P ≤0.05 level. Comparisons were made between normal and high vitamin A-fed groups of each phenotype (A I-Lean: 2.6 mg vitamin A/kg diet, A II-Lean: 129 mg vitamin A/kg diet, B I-Obese: 2.6 mg vitamin A/kg diet and B II-Obese: 129 mg/g diet).

To explain the possible mechanism involved in the vitamin-A mediated downregulation of 11β-HSD1 in liver, we studied the hepatic expression of C/EBPα and LXRα both at mRNA and protein levels. Vitamin A supplementation significantly increased the hepatic C/EBPα gene expression in both the phenotypes (Figure 3) as compared with their respective control rats. However, elevated hepatic C/EBPα protein levels were observed only in vitamin A- supplemented lean rats, where as in obese rats, hepatic C/EBPα protein levels were significantly low (Figure 3). Hepatic LXRα mRNA and protein levels significantly decreased by feeding of vitamin A enriched diet to WNIN/Ob lean and obese rats as compared with their respective stock diet fed-lean and obese counterparts (Figure 4).

### Effect of vitamin A supplementation on hepatic glycerol-3-phospahte dehydrogenase (GPDH) activity

**Figure 5:**
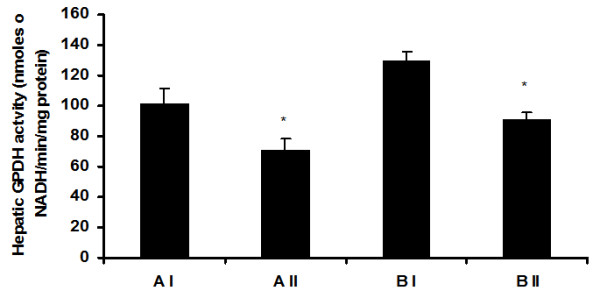
**Effect of vitamin A supplementation on hepatic GPDH activity in WNIN/Ob lean and obese rats**. Activity was reported as nmoles of NADH utilized/min/mg protein. Values are means ± SE for 8 rats at P ≤0.05 level. Comparisons were made between normal and high vitamin A-fed groups of each phenotype (AI-Lean: 2.6 mg vitamin A/kg diet, AII-Lean: 129 mg vitamin A/kg diet, BI-Obese: 2.6 mg vitamin A/kg diet and BII-Obese: 129 mg/kg diet).

As vitamin A supplementation decreased hepatic 11β-HSD1 activity in lean and obese rats, we studied the activity of cytosolic glycerol-3-phosphate dehydrogenase (GPDH), which is induced by glucocorticoids in hepatocytes (17). In line with the decreased 11β-HSD1 activity, GPDH activity was also significantly decreased by vitamin A supplementation in both the phenotypes as compared with their respective controls (Figure 5).

## Discussion

In this communication, we report the impact of chronic feeding of vitamin A-enriched diet on 11β-HSD1 activity in liver and visceral fat of WNIN/Ob obese rats, a new genetic rat model of obesity. Here, we demonstrate that feeding of vitamin A-enriched diet to WNIN/Ob obese rats decreases body weight, fat mass and 11β-HSD1 activity in liver and visceral fat. To our knowledge, this is the first study to link vitamin A and pre-receptor metabolism of glucocorticoids in normal and obese conditions.

Retinoic acid, one of the metabolically-active forms of vitamin A, regulates cellular gene expression through retinoic acid receptors (RARs) and retinoid × receptors (RXRs). Adipose tissue is a good target organ for vitamin A action, as it stores significant amount of vitamin A and expresses RAR and RXR transcription factors. Retinoic acid is known to inhibit preadipocyte differentiation [18] and remodel white adipose tissue to brown adipose tissue [19]. Retinaldehyde, another functional form of vitamin A is reported to decrease adipogenesis and ameliorate diet-induced obesity in rodent models [20]. In line with our previous observation, high (but not toxic) dose of vitamin A decreased visceral fat mass and bodyweight in obese rats [10]. Previous studies have reported that cortisol (corticosterone in rodents) is essential for preadipocyte differentiation [21] and adipose-specific overexpression of 11β-HSD1 in mice, results in increased preadiocyte differentiation [3]. As reported previously, WNIN/Ob obese rats have exhibited higher 11β-HSD1 activity in visceral fat than their lean counter parts [22]. In the present study, vitamin A significantly decreased 11β-HSD1 activity in visceral fat, which is associated with significant decrease in visceral fat mass. Vitamin A mediated visceral fat loss in obese rats could be due to decreased preadipocyte differentiation, as reduced 11β-HSD1 activity results in decreased tissue corticosterone levels. In contrast to the observation in obese rats, vitamin A-challenged lean rats had increased 11β-HSD1 activity in visceral fat, however, increased enzyme activity had no impact on visceral fat mass. The differential effects of vitamin A on 11β-HSD1 activity and visceral fat mass in lean and obese rats could be due to other physiological factors that are altered in obese condition.

Vitamin A may regulate the 11β-HSD1 activity, by altering its gene expression through direct and indirect mechanisms. Presently, retinoic acid-response elements are not reported on 11β-HSD1 promoter to support the direct effect of vitamin A on 11β-HSD1 gene expression. C/EBPα has been shown to be obligatory for the expression of 11β-HSD1 [4]. Previous studies have reported that retinoic acid downregulates the expression of genes like resistin that are regulated by C/EBPα in adipose tissue [13]. Vitamin A-mediated regulation of 11β-HSD1 gene expression in adipose tissue may be mediated by indirect mechanism through C/EBPα.

11β-HSD1 plays an important role in the regulation of carbohydrate and lipid metabolism in liver. 11β-HSD1-KO mice have improved insulin sensitivity [4], where as transgenic overexpression of 11β-HSD1 in liver results in the development of impaired glucose tolerance [23]. WNIN/Ob obese rats have lower hepatic 11β-HSD1 expression and activity as observed in other obese rodent models [22]. In the present study, vitamin A supplementation at high doses decreased hepatic 11β-HSD1 activity and expression in both lean and obese phenotypes. Supporting to our hypothesis, activity of GPDH, a glucocorticoid inducible enzyme, was also decreased in vitamin A supplemented lean and obese rats. Based on these observations, it is possible that vitamin A may regulate the expression of hepatic glucocorticoid target genes by regulating the expression of 11β-HSD1.

To understand the possible mechanisms involved in the downregulation of 11β-HSD1 in liver, we studied the expression of C/EBPα at mRNA and protein levels. In our study, vitamin A supplementation increased hepatic C/EBPα gene expression in vitamin A-treated lean and obese rats. In contrast to increased mRNA levels, C/EBPα protein levels decreased in vitamin A-supplemented obese rats, suggesting the role of post-transcriptional regulatory mechanisms. Possibly, vitamin A-mediated downregulation of hepatic 11β-HSD1 is mediated through different mechanisms in lean and obese rats, in other words, vitamin A-mediated down regulation of hepatic 11β-HSD1 may be C/EBPα-dependent in obese rats, while it is independent of C/EBPα in lean rats.

Previous studies have reported that LXRα ligands down regulate 11β-HSD1 through indirect mechanisms [7]. In this study, vitamin A supplementation increased hepatic LXRα mRNA and protein levels in WNIN/Ob lean and obese rats. In support to the elevated LXRα gene expression, ABCA1 (ATP-binding cassette transporter protein 1), a classical target gene of LXRα is elevated in lean and obese rats (unpublished data). Vitamin A-mediated downregulation of hepatic 11β-HSD1 gene expression is may be mediated through LXRα. Another mechanism, through which dietary vitamin A may down regulate 11β-HSD1, is by providing high concentration of RXR ligand, 9-cis-retinoic acid. LXRα forms heterodimer with RXR and this step is essential for LXRα-mediated gene transcription. Higher concentration of RXR ligand may result in the increased recruitment of LXRα:RXR heterodimers to 11β-HSD1 gene promoter resulting in decreased 11β-HSD1 gene expression.

11β-HSD1 gene expression in liver and adipose tissue is also regulated by various hormones and cytokines present in the plasma [24]. It is possible that Vitamin A-mediated alterations in the levels of these signaling molecules may also affect the 11β-HSD1 activity in liver and adipose tissue of WNIN/Ob lean and obese rats.

## Conclusions

In summary, we showed for the first time that supra-physiological dose of vitamin A through diet decreases 11β-HSD1 activity in visceral fat and liver of WNIN/Ob obese rat. The observed vitamin A-mediated reduction in 11β-HSD1 activity in the visceral fat of obese rats may contribute to the decreased visceral fat mass in this model. Further research is needed to understand the mechanisms involved in the regulation of 11β-HSD1 by various nutrients in tissues like liver and visceral fat in order to develop appropriate dietary interventions to prevent the development of obesity and insulin resistance.

## List of abbreviations used

11β-HSD1: 11β-hydroxysteroid dehydrogenase type 1; C/EBPα: CCAAT/Enhancer-binding protein α; LXRα: Liver × receptor α; RXR: Retinoid × receptor; RAR: Retinoic acid receptor; HPLC: High-performance liquid chromatography; SDS-PAGE: Sodium dodecyl sulphate- polyacrylamide gel electrophoresis.

## Competing interests

The authors declare that they have no competing interests.

## Authors' contributions

SSSVP carried out plasma corticosterone and 11β-HSD1 assays, gene expression work, data analysis and prepared the first draft. PA carried out the animal experiment, immunoblotting work, estimated retinol, GPDH activity, data analysis and manuscript editing. GNV was responsible for breeding and supply of animals. VA hypothesized, designed and supervised the experiment. VA reviewed, finalized the draft and also gave permission to submit manuscript. All authors read and approved the content of the manuscript.
